# *Leishmania* Metacyclogenesis Is Promoted in the Absence of Purines

**DOI:** 10.1371/journal.pntd.0001833

**Published:** 2012-09-20

**Authors:** Tiago Donatelli Serafim, Amanda Braga Figueiredo, Pedro Augusto Carvalho Costa, Eduardo Almeida Marques-da-Silva, Ricardo Gonçalves, Sandra Aparecida Lima de Moura, Nelder Figueiredo Gontijo, Sydnei Magno da Silva, Marilene Suzan Marques Michalick, José Roberto Meyer-Fernandes, Roberto Paes de Carvalho, Silvia Reni Bortolin Uliana, Juliana Lopes Rangel Fietto, Luís Carlos Crocco Afonso

**Affiliations:** 1 Laboratório de Imunoparasitologia, Departamento de Ciências Biológicas, Núcleo de Pesquisas em Ciências Biológicas, Universidade Federal de Ouro Preto, Ouro Preto, Brazil; 2 Laboratório de Imunopatologia, Departamento de Ciências Biológicas, Núcleo de Pesquisas em Ciências Biológicas, Universidade Federal de Ouro Preto, Ouro Preto, Brazil; 3 Laboratório de Fisiologia de Insetos Hematófagos, Departamento de Parasitologia/ICB, Universidade Federal de Minas Gerais, Belo Horizonte, Brazil; 4 Laboratório de Sorologia, Departamento de Parasitologia/ICB, Universidade Federal de Minas Gerais, Belo Horizonte, Brazil; 5 Laboratório de Bioquímica Celular, Instituto de Bioquímica Médica, Universidade Federal do Rio de Janeiro, Rio de Janeiro, Brazil; 6 Laboratório de Neurobiologia Celular, Departamento de Neurobiologia/Programa de Neurociências, Universidade Federal Fluminense, Niterói, Brazil; 7 Departamento de Parasitologia, Instituto de Ciências Biomédicas, Universidade de São Paulo, São Paulo, Brazil; 8 Laboratório de Infectologia Molecular Animal, Departamento de Bioquímica e Biologia Molecular/Bioagro, Universidade Federal de Viçosa, Viçosa, Brazil; Lancaster University, United Kingdom

## Abstract

*Leishmania* parasites, the causative agent of leishmaniasis, are transmitted through the bite of an infected sand fly. *Leishmania* parasites present two basic forms known as promastigote and amastigote which, respectively, parasitizes the vector and the mammalian hosts. Infection of the vertebrate host is dependent on the development, in the vector, of metacyclic promastigotes, however, little is known about the factors that trigger metacyclogenesis in *Leishmania* parasites. It has been generally stated that “stressful conditions” will lead to development of metacyclic forms, and with the exception of a few studies no detailed analysis of the molecular nature of the stress factor has been performed. Here we show that presence/absence of nucleosides, especially adenosine, controls metacyclogenesis both *in vitro* and *in vivo*. We found that addition of an adenosine-receptor antagonist to *in vitro* cultures of *Leishmania amazonensis* significantly increases metacyclogenesis, an effect that can be reversed by the presence of specific purine nucleosides or nucleobases. Furthermore, our results show that proliferation and metacyclogenesis are independently regulated and that addition of adenosine to culture medium is sufficient to recover proliferative characteristics for purified metacyclic promastigotes. More importantly, we show that metacyclogenesis was inhibited in sand flies infected with *Leishmania infantum chagasi* that were fed a mixture of sucrose and adenosine. Our results fill a gap in the life cycle of *Leishmania* parasites by demonstrating how metacyclogenesis, a key point in the propagation of the parasite to the mammalian host, can be controlled by the presence of specific purines.

## Author Summary

*Leishmania* parasites are the causative agent of a spectrum of diseases characterized by severe lesions in skin or life threatening visceral infections. In the parasite life cycle, a range of morphological transitions can be found such as amastigotes (hosted in humans and others mammals) and promastigotes (located in the sand fly vector). The disease begins when the infective non-dividing promastigote metacyclic form is transmitted to mammalian hosts by the bite of an infected sand fly. The conditions for the development of these metacyclic promastigotes are still not fully understood. Here we tested the hypothesis that the presence or absence of purine will determine whether the parasite will proliferate or differentiate into the metacyclic form. Our experiments indicate that the presence of purines in the culture medium of *Leishmania* parasites interfere with the development of metacyclic promastigotes. Our results also show that although decreased proliferation and metacyclic differentiation occur simultaneously these two phenomena are independently regulated. Finally we show, for the first time in a natural vector, that metacyclogenesis is inhibited *in vivo* by adenosine, suggesting a new level of cell differentiation control in these protozoan parasites which is driven by purine sensing.

## Introduction

Protozoan parasites from *Leishmania* genus are the causative agents of leishmaniasis, a broad spectrum disease that range from asymptomatic infections to disfiguring forms such as diffuse or mucosal leishmaniasis as well as visceral leishmaniasis, which can be fatal if not adequately treated. The outcome of this infection in humans depends largely on the immune response assembled by the host and the virulence and species of the parasite.

Basically developmental stages of these protozoa alternate between amastigotes that live in mammalian macrophages and generate the disease manifestations mentioned above, and promastigotes which are present in the midguts of female sand flies. During its life cycle in the invertebrate host, *Leishmania* promastigotes undergo a series of morphological changes that culminate with the differentiation into the metacyclic form, which is responsible for the initiation of infection in the vertebrate host. Although this developmental stage has been described for nearly 30 years [1], the factors that trigger metacyclogenesis in *Leishmania* parasites are still poorly understood. It has been generally stated that “stressful conditions” will lead to development of metacyclic forms and with the exception of a few studies no detailed analysis of the molecular nature of the stress factor has been performed [2]. Based mainly in *in vitro* studies, it has been demonstrated that low pH, lack of nutrients and low levels of tetrahydrobiopterin influence metacyclogenesis [1], [3], [4]. However, no specific role of these factors *in vivo* has ever been confirmed.

*Leishmania* and other trypanosomatids are unable to synthesize the purine ring by the *de novo* pathway and depend on the uptake of nucleosides and nucleobases to supply the purine salvage pathways [5]. The present study reports that metacyclogenesis induction is controlled by the presence of adenosine. We observed that addition of CGS 15943 (CGS), a potent antagonist of mammalian adenosine receptors [6], strongly induces metacyclogenesis in promastigote cultures. We also show that although CGS interferes with the transport of adenosine by the parasite, induction of metacyclogenesis cannot be attributed to lack of precursors for the purine salvage pathway and does not correlate with lack of parasite proliferation. Furthermore, we show that addition of adenosine to cultures of metacyclic promastigotes induces differentiation of these cells into proliferative stages of the parasite. Finally, we show that the presence of adenosine in the sugar meal of infected sand flies inhibit metacyclogenesis indicating that the effect of adenosine on metacyclogenesis is not restricted to the development of the parasite *in vitro*.

## Methods

### Ethics statement

The protocols to which animals were submitted were approved by the Universidade Federal de Ouro Preto (OFíCIO CEP N°. 005/2009) and by the Universidade Federal de Minas Gerais Ethical Committees on Animal Experimentation (125/05 and 211/07) and followed the guidelines from the Canadian Council on Animal Care.

### Parasites

*Leishmania amazonensis* [PH8 strain (IFLA/BR/67/PH8)] and *Leishmania major* [FRIEDLIN strain (MHOM/IL/80/Friedlin)] were cultured in Grace's insect medium (Sigma Aldrich) supplemented with 10% heat-inactivated fetal calf serum (FCS; LGC Biotecnologia), 2 mM L-glutamine (GIBCO BRL) and 100 U/ml penicillin G potassium (USB Corporation), pH 6.5, at 25**°**C. Parasites were sub-cultured at 1×10^5^ parasites/ml 3 days before experiments in order to achieve a mid-log phase. Throughout this work, promastigotes cultures were initiated with 2×10^6^ cells/ml and incubated for 3 days. Parasites cultures were initiated from frozen stocks (first passage) obtained after the transformation of C57BL/6 mice lesion amastigotes. In vitro cultures of differentiated promastigotes, for all experiments, were maintained in conditions described above for a maximum of ten passages. This limit of passages is adopted to prevent loss of metacyclic characteristics, such as virulence, as previous described [7].

### Viability analysis of promastigotes

Viability of parasites was assessed by flow cytometry using propidium iodide incorporation [8]. Parasites were washed twice, resuspended at 2×10^6^/ml in PBS and, at the moment of acquisition, 5 µl/ml of a propidium iodide staining solution (eBioscience) was added to samples. Data were collected in BD FACSCalibur flow cytometer. Cell acquisition was performed using BD CellQuest Pro software and data analysis was performed using FlowJo software ver. 9.4 (Tree Star, Inc). Fifth thousand events were harvested from each sample.

### Animals

Female BALB/c and C57BL/6 mice (4–8 weeks old) were obtained from the University's animal facility (CCA - UFOP). A mongrel dog of unknown age naturally infected with *Leishmania infantum chagasi* from an endemic area of visceral leishmaniasis in Minas Gerais State (southeast Brazil) was housed in a kennel at Universidade Federal de Minas Gerais animal facility. Animals were given water and food *ad libitum*. Adult females of *Lutzomyia longipalpis* were maintained in a closed colony in UFMG as described [9].

### Metacyclogenesis induction

Unless otherwise stated in figure legends, CGS 15943 (Tocris Bioscience/Sigma-Aldrich) (50 µM) was added to culture after 48 hr of growth and metacyclic promastigotes quantified after 24 hr. In experiments of metacyclogenesis induction, nucleosides [adenosine (0.5 mM), inosine (0.5 mM)], nucleobases [adenine (0.5 mM) and hypoxanthine (0.1 mM)], dipyridamole (0.05 or 0.1 mM) and N^6^-methyladenine (2 mM) were also added alone or with CGS (see figure legends) after 48 hr of culture and incubated for 24 hr. CGS 15943, Dipyridamole, N^6^-Methyladenine and hypoxanthine were prepared in DMSO (LGC Biotecnologia; 1–3% final concentration).

### Metacyclogenesis evaluation by morphology

Morphological evaluation of metacyclogenesis was performed under light microscopy. Parasites were considered metacyclic when presenting small body cell size and long flagellum (twice or more the body length).

### Metacyclic promastigotes enrichment by Ficoll density gradient

Metacyclic promastigotes were enriched by centrifugation of promastigotes over Ficoll 400 (Sigma-Aldrich) gradient and quantified by hemocytometer counting [10], [11]. Results reflect the total number of parasites obtained from the gradient. In general the percentage of metacyclic promastigotes in the enriched fraction was greater than 80%.

### Complement-mediated lysis of promastigotes

Promastigotes obtained from control or CGS-treated cultures were incubated in Hank's balanced salt solution prepared with 1 mM MgCl_2_ and 0.15 mM CaCl_2_ in the presence of 10% of fresh rat serum. After growing parasites as mentioned above, cells were washed twice and resuspended at 1×10^8^/ml in HBSS, pH 7.4 plus 10% rat serum and incubated at 37°C for 1 h. Reaction was stopped diluting the samples 100-fold with ice-cold HBSS and centrifuge cells at 1540× *g*/10 min/4°C. Parasite survival was assessed by counting whole cells on a hemocytometer. These procedures were adapted from [12].

### Western blotting for META-1 protein

Total protein extracts were prepared [13] and samples equivalent to 2×10^7^ promastigotes were fractionated in 15% SDS-PAGE and transferred to nitrocellulose membranes. Immunoblotting was performed as described [13] with anti-META1 polyclonal antiserum diluted 1∶500. Membranes were developed using chemiluminescent substrate (SuperSignal, Thermo Scientific).

### Attachment to sand fly midgut

Promastigotes were washed twice with 0.9% NaCl solution and adjusted to 2×10^7^ cells/ml. Intestines of adult females of *Lutzomyia longipalpis*, maintained as described [9], were dissected (10 per group), and posterior portion and Malpighi tubules were removed. After this, midguts were opened and placed into 30 µL 0.9% NaCl solution plus 1% hemoglobin in a scooped glass slide (Sigma Aldrich). Hemoglobin 0.5% final concentration was used to prevent the parasite attachment to the glass. 30 µL of *L. amazonensis* suspension were added to midguts and incubated for 35 min/25°C. After washing twice with saline, midguts were homogenized with a teflon pestle in micro-centrifuge conical tubes and fixed on slides for Giemsa staining. Midgut homogenates were evaluated for total number of promastigotes by optical microscopy. These procedures were adapted from [14].

### *In vitro* infectivity assay

Thioglycolate elicited peritoneal cells were harvested and plated (1×10^6^ cells) onto round cover-slips in supplemented DMEM in 24-well plates and rested overnight at 37**°**C, 5% CO_2_. Non-adherent cells were removed by washing with warm PBS. Parasites were added for 3 hr (5 parasites per macrophage), washed and incubated for another 72 hr at 37°C/5% CO_2_. After 72 hr of infection, coverslips were fixed in methanol for 2 min (Vetec Fine Chemistry), and stained using the kit Panótico Rápido (Renilab) - a Romanowsky like stain, according to manufacturer's instructions for the assessment of cellular parasitism. The analysis was performed using an Olympus BX50 optical microscope. The number of infected and uninfected cells was determined in a minimum of 100 macrophages per coverslip.

### *In vivo* infectivity assay

C57BL/6 mice were inoculated in the left ear with 1×10^3^ promastigotes/10 µL PBS originated from control or CGS-treated cultures. Lesion development was followed weekly with a digital caliper (Starrett, model 727). The results were expressed as the difference between measures of infected and contralateral non-infected ears.

### Parasite load assessment

The number of parasites in the ear was estimated by a limiting dilution assay [11], [15]. After seven weeks of infection, mice were euthanized and the ears collected, the ventral and dorsal dermal sheets separated and incubated, dermal side down, in RPMI-1640 medium, pH 7.2 (Sigma-Aldrich) with collagenase A (1 mg/mL) (Sigma-Aldrich) for 2 hr at 37°C/5% CO_2_. Ears were ground in Grace's insect medium, pH 6.5, in a glass tissue grinder. Tissue debris was removed by centrifugation at 50× *g*/4°C/1 min and supernatant transferred to another tube and centrifuged at 1540× *g*/4°C/15 min. The pellet was resuspended in 0.5 ml Grace's insect medium supplemented with 10% heat-inactivated FCS, 2 mM L-glutamine and 100 U/ml penicillin G potassium, pH 6.5. Parasite suspension was serially diluted in 10-fold dilutions in duplicates to a final volume of 200 µl in 96-well plates. Pipette tips were replaced for each dilution. Plates were incubated for 15 days at 25°C and examined under an inverted microscope for the presence of parasites. Results were expressed as log of the last dilution in which they were detected.

### Adenosine uptake by *L. amazonensis*

Uptake of [3H]adenosine by *L. amazonensis* promastigotes was assayed as [16], [17] with few modifications. Promastigotes from middle log phase cultures were washed twice in buffer (116 mM NaCl, 5.4 mM KCl, 5.5 mM glucose, Hepes/Tris 30 mM, pH 7.4) and resuspended at 5×10^8^ cells/ml in the same buffer. Parasites were incubated in for 20 min with or without CGS 15943 or dipyridamole (10, 50 or 100 µM). Transport was measured at 25°C and initiated by adding 100 µL of cells to 100 µL of radiolabeled adenosine at 2 µM/0.2 µCi, diluted in buffer containing CGS 15943 or dipyridamole at the concentrations described above. After 60 s, transport was stopped by spinning the cells (10000× *g*/120 s) through an oil cushion of 100 µl of dibutyl phthalate (Sigma-Aldrich). Aqueous and oil phase were removed and pellet dissolved with 2% Triton X-100 (Sigma-Aldrich). Lysate was mixed with 2 ml of scintillation solution (Optiphase HiSafe 3 - PerkinElmer) for liquid scintillation counting (Tri-Carb 2810 TR - PerkinElmer).

### Evaluation of metacyclogenesis *in vivo*

To evaluate metacyclogenesis *in vivo*, adult insects of *Lutzomyia longipalpis*, majority of females, were allowed to feed in a naturally *Leishmania infantum chagasi*-infected dog for 30 min. Insects were fed for 8 to10 days with 30% sucrose solution with or without adenosine (5 mM). Midguts were dissected, homogenized individually with a teflon pestle in micro-centrifuge conical tubes and fixed on slides for Giemsa staining. Each midgut was evaluated for the presence of metacyclic promastigotes by microscopy.

### Statistical analysis

Student's *t*-test was performed using Prism 5.0 software (GraphPad Software). p<0.05 was considered statistically significant.

## Results and Discussion

**Figure 1:**
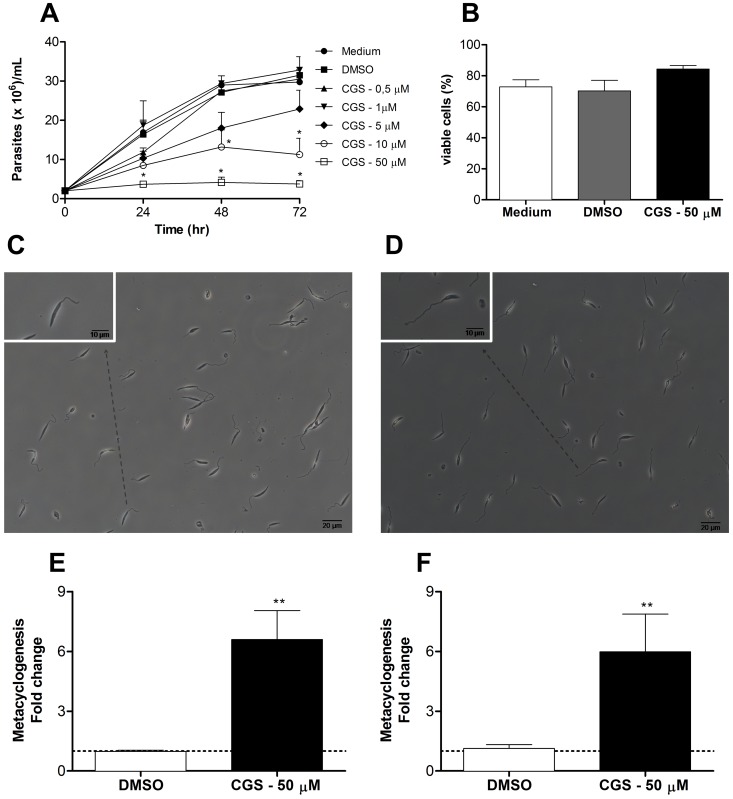
Presence of CGS 15943 in *Leishmania amazonensis* culture halts parasite growth and enhances metacyclogenesis. (A) Evaluation of *L. amazonensis* growth in Grace's insect medium containing different concentrations of CGS as determined by hemocytometer counting. (B) Viability of parasites was assessed by flow cytometry using propidium iodide incorporation after 72 hr of culture. Phase contrast image of DMSO-treated (C) and CGS-treated (D) cultures of *L. amazonensis* promastigotes (picture taken with an Axio Cam MR3 attached to a Carl-Zeiss Axio Imager M2 microscope). Percentage of metacyclic promastigotes in control (Medium; DMSO) and CGS-treated cultures was evaluated by differential counting in optical microscopy (E) and Ficoll density gradient purification (F). Means and standard deviations from at least three independent experiments are plotted; *p<0.05, **p<0.01 determined by two-tailed Student's t-test indicate significant difference from control group.

Our laboratory has been involved in the study of the role of adenosine receptors in the control of inflammatory response in a murine model of cutaneous leishmaniasis and has been interested in the effect of antagonists of these receptors on the course of infection by *L. amazonensis*. Thus, in order to exclude a possible direct effect of the receptor antagonist on parasite growth, promastigotes of *L. amazonensis* were cultured in the presence of CGS. Interestingly, the addition of CGS to culture medium prevented parasite growth when added in concentrations higher than 10 µM (Figure 1A) without, however, interfering with parasite viability (Figure 1B). The effect of CGS on parasite growth was reversible and also independent of the time when the drug was added to the culture (Figure S1). We also observed that cultures of CGS-treated parasites presented an increased proportion of highly motile cells with short body length and relatively long flagella, characteristic of the infective metacyclic promastigotes [18], [19] (Figures 1C–D and Video S1, Video S2, Video S3, Video S4). Parasite species and culture conditions (medium composition, temperature, culture phase [log versus stationary] and parasite density) influence significantly the development of metacyclic promastigotes with optimum yield ranging usually from 1 to 5% [20]. In this study, assessment of cultures after 72 hr of growth, both by differential counting and by density gradient centrifugation [10], demonstrated a six fold increase in the percentage of metacyclic promastigotes in CGS-treated cultures (6 to 25%) when compared to control cultures (1 to 3%) (Figures 1E–F).

**Figure 2:**
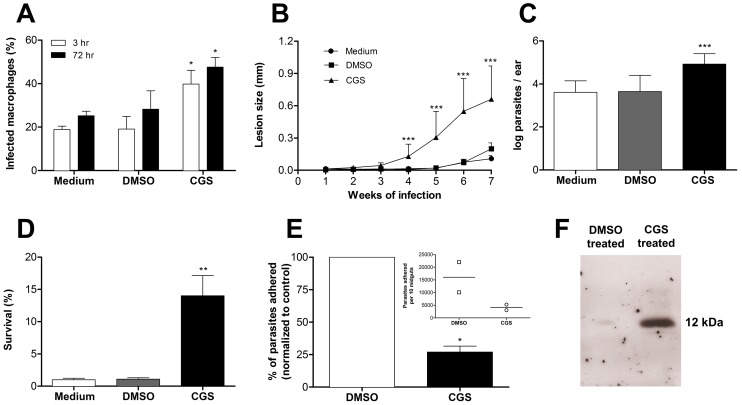
Functional evaluation of metacyclogenesis in *Leishmania amazonensis* cultures. (A) Percentage of infected peritoneal macrophages. Asterisks indicate significant difference as compared to the respective control groups (3 and 72 hr). (B) Lesion development in C57BL/6 mice injected in the ear dermis with 1×10^3^ promastigotes. After 7 weeks of infection, parasite burden was evaluated by limiting dilution assay (C). (D) Sensitivity of parasites to complement-mediated lysis. Promastigotes were incubated for 1 h/37°C in HBSS plus10% fresh rat serum. Percentage of survival was determined by counting intact cells in a hemocytometer. (E) Percentage of promastigotes bound to female *Lutzomyia longipalpis* midguts after incubation for 35 min. Inset shows the total number of adhered promastigotes per 10 midguts. (F) Western blotting for META-1 protein. Total protein extracts (equivalent to 2×10^7^ promastigotes) were fractionated in 15% SDS-PAGE and transferred to nitrocellulose membranes. Immunoblotting was performed anti-META1 polyclonal antiserum. Means and standard deviations from at least three independent experiments (except for “E” with 2 experiments) are plotted; *p<0.05, **p<0.01, ***p<0.0001 determined by two-tailed Student's t-test indicate significant difference from control groups.

Since the morphological aspects of metacyclogenesis in *Leishmania* promastigotes are subtle and possibly subjective, we decided to further evaluate the increased induction of metacyclogenesis by CGS by analyzing functional characteristics of this developmental stage. *Leishmania* metacyclic promastigotes differ from other developmental stages in several aspects such as infectivity, resistance to complement-mediated lysis and attachment to sand fly midgut epithelium [1], [14], [21]–[23]. Thus, stationary phase promastigotes from CGS-treated cultures were added to BALB/c mice peritoneal macrophages cultures. Our results show that promastigotes from CGS-treated cultures were more infective to macrophages than control (medium or DMSO- treated) cultures, both at 3 and 72 hr of infection (Figure 2A). More importantly, C57BL/6 mice inoculated in the ear with 1×10^3^ stationary phase promastigotes from CGS-treated cultures developed larger lesions and presented higher tissue parasitism (Figures 2B–C).

Metacyclogenesis in *Leishmania* parasites has been associated with increased expression of a major surface proteinase (gp63) and modifications of the molecular structure of a surface lipophosphoglican (LPG), both of which contribute to increased resistance to complement-mediated lysis that allows for extended survival of the promastigote form within the vertebrate host prior to its uptake by phagocytes [24], [25]. Our results (Figure 2D) show that CGS-treated stationary phase *L. amazonensis* promastigotes were significantly more resistant to complement mediated lysis than promastigotes from control cultures.

In the insect vector, *Leishmania* promastigotes go through a series of developmental stages that are associated with different ability to adhere to microvilli of the insect midgut epithelium [4], [26]. This differential attachment to the midgut has been attributed to structural changes in the LPG molecule especially in the composition of terminal sugar residues [27]. It is believed that changes that occur in the LPG during metacyclogenesis are responsible for the detachment of the infective forms of the parasite allowing its transmission to the mammalian host during blood feeding [4]. We evaluated the *in vitro* attachment of stationary phase promastigotes to the midgut of *Lutzomyia longipalpis*. Although *Lutzomyia longipalpis* is not the natural vector of *L. amazonensis*, it has been shown to support infection by different species of *Leishmania* [28]–[30]. Our results show a significant decrease in attachment to the insect midgut in CGS-treated promastigotes, indicating a higher proportion of metacyclic forms in these cultures (Figure 2E). Finally, parasites from CGS-treated cultures present higher expression of the META-1 protein (Figure 2F), characteristic of metacyclic promastigotes [13]. Altogether, these results indicate that the morphological changes observed in CGS-treated cultures (Figure 1) are consistent with the development of a higher proportion of promastigotes with the same characteristics of those described as metacyclic promastigotes in the studies that initially identified this developmental stage of the parasite and others that further characterized this population [1], [18], [21], [31]. The different levels of metacyclogenesis indicated by the several assays presented here may be related to the fact that each protocol analyses a different aspect of the phenomenon that may not occur concomitantly during parasite development.

CGS 15943 acts as an antagonist of all four known mammalian adenosine receptors. However, no adenosine receptor has been described for *Leishmania* or other trypanosomatids. Also, we exhaustively searched for proteins homologous to the mammalian adenosine receptors in *Leishmania* genomes databank and no significant similarity was found. On the other hand, several nucleoside and nucleobase transporters have been characterized in *Leishmania major* and *Leishmania donovani* [17], [32], [33] which are important to the parasite's metabolism given the fact that trypanosomatids do not present the enzymes for the *de novo* synthesis of purines [34], [35].

**Figure 3:**
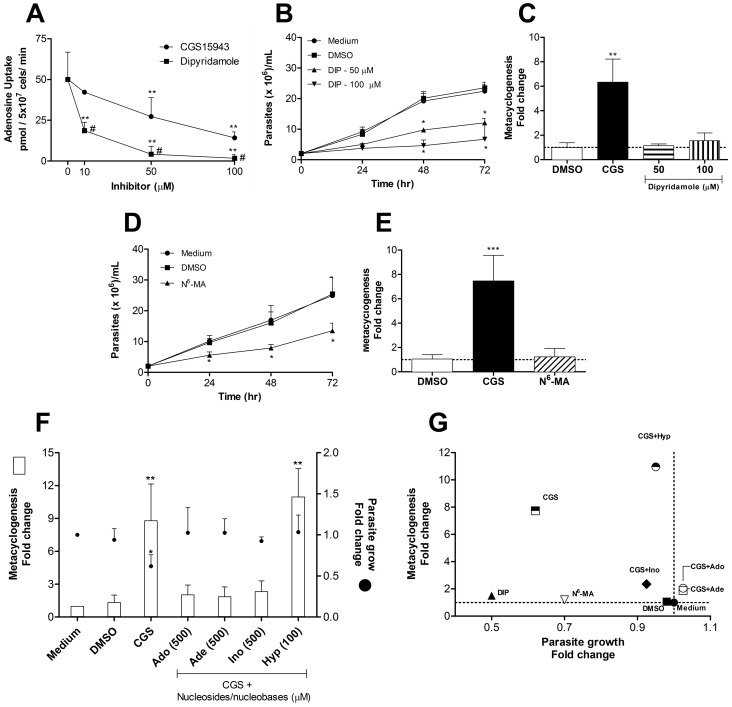
Control of metacyclogenesis *in vitro*. (A) Uptake of [^3^H]adenosine by mid-log phase promastigotes of *L. amazonensis* in presence of CGS or dipyridamole (DIP). Experiments were performed at pH 7.4. # indicates p<0.01 when we compared DIP with CGS groups using the two-tailed Student's t-test. (B) *L. amazonensis g*rowth curve and metacyclogenesis (C) in the presence of DIP (please see Figure 1 for details). (D) Growth evaluation of *L. amazonensis* in presence of N^6^methyladenine (N^6^-MA) followed by metacyclogenesis assessment (E). (F) Assessment of metacyclogenesis in cultures stimulated with CGS and CGS plus adenosine (Ado), inosine (Ino), adenine (Ade) or hypoxanthine (Hyp). CGS was always used at concentration of 50 µM and for nucleosides/nucleobases, the concentration is indicated in parentheses (µM). Experiments were performed in Grace's culture medium supplemented with fetal bovine serum, glutamine and penicillin, pH 6.5. (G) Compilation of results from experiments evaluating growth curve and metacyclogenesis in different conditions. In all experiments metacyclogenesis was evaluated by Ficoll density gradient. Means and standard deviations from at least three independent experiments are plotted; *p<0.05, **p<0.01 determined by two-tailed Student's t-test indicate significant difference from control group.

Our first approach in order to understand CGS effects was to evaluate its possible role on adenosine uptake. As shown in Figure 3A, dipyridamole, a known inhibitor of adenosine transport [36], [37], inhibited adenosine transport in a dose-dependent manner. On the other hand, incubation of *L. amazonensis* promastigotes with the same concentrations of CGS led to a smaller although significant inhibition of adenosine uptake by the parasite. Interestingly, however, although addition of dipyridamole to promastigote cultures inhibited parasite growth (Figure 3B), it had no effect on the induction of metacyclogenesis (Figure 3C). Thus, while CGS effects on parasite proliferation might be attributed, at least in part, to inhibition of adenosine uptake with consequent inhibition of purine nucleotide biosynthesis, induction of metacyclogenesis does not seem to be associated with diminished uptake of this nucleoside. To confirm that proliferation rather than metacyclogenesis is associated with purine nucleotide starvation, we incubated *L. amazonensis* promastigotes with an inhibitor of enzymes that participate in the purine salvage pathways. As shown in Figure 3D, addition of N^6^-methyladenine, an inhibitor of guanine and adenosine deaminase [38], significantly inhibited parasite proliferation *in vitro* without, however, interfering with metacyclogenesis (Figure 3E). These results strongly suggest that interference with the parasite's ability to proliferate does not, necessarily, implicate in differentiation into metacyclic forms. This hypothesis was further confirmed by the addition of hypoxanthine (a central intermediate of the purine salvage pathway) to CGS-treated cultures. Addition of adenosine, adenine or inosine to CGS-treated cultures abolished the inhibitory effect of CGS on parasite proliferation and simultaneously reduced metacyclogenesis. On the other hand, addition of hypoxanthine was able to reverse CGS-induced inhibition of parasite growth without, however, interfering with metacyclogenesis induction (Figure 3F). Due to its low solubility hypoxanthine was used at a lower concentration than the other purines. However, even at a lower concentration hypoxanthine was able to completely recover parasite growth and did not interfere with metacyclogenesis induction. As expected, addition of purines to parasite cultures in the absence of CGS resulted in enhanced parasite proliferation and decreased metacyclogenesis (Figure S2).

Taken together these results indicate that, contrary to previous belief, inhibition of parasite growth by lack of nutrients is not necessarily associated with differentiation to metacyclic forms. As shown in Figure 3G, no correlation between parasite growth and induction of metacyclogenesis is observed under the conditions tested in this study.

**Figure 4:**
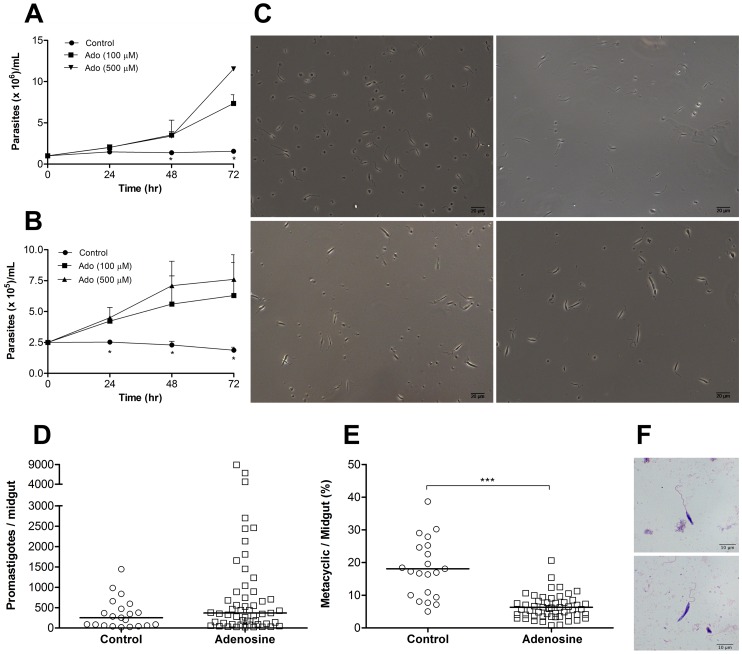
Adenosine reverses metacyclogenesis *in vitro* and reduces *in vivo* development of metacyclic promastigotes. (A) Metacyclic promastigotes from CGS-treated cultures, obtained by Ficoll gradient centrifugation, were cultivated in “spent” medium (late stationary phase medium from control cultures) or in “spent” medium plus adenosine (Ado). Parasite growth was evaluated by hemocytometer counting. (B) Metacyclic promastigotes from CGS-treated cultures, obtained by Ficoll gradient centrifugation followed by complement mediated lysis (as in Figure 2D), were cultured in Grace's insect medium without supplements or in this medium plus adenosine. Parasite growth was evaluated by hemocytometer counting. (C) General aspect of metacyclic promastigotes after double purification (Ficoll + complement) (upper left). Cells after 72 hr of incubation in Grace's insect medium without supplements (upper right), or in the presence of 100 µM (lower left) or 500 µM of adenosine (lower right). Pictures taken with an Axio Cam MR3 attached to a Carl-Zeiss Axio Imager M2 microscope. Adult insects of *Lutzomyia longipalpis*, majority of females, were allowed to feed in a naturally *Leishmania infantum chagasi*-infected dog for 30 min. Insects were fed for 8 to10 days with 30% sucrose solution with or without adenosine (5 mM). Each midgut was evaluated by light microscopy for the total amount of parasites (D) and for the percentage of metacyclic promastigotes (E). (F) Image of a metacyclic (upper panel) and non-metacyclic promastigote (lower panel) of *L. infantum chagasi* from sand fly midgut. Pictures taken with a DFC300FX camera attached to a Leica DM5000B microscope. Means and standard deviations (or medians in graph E) from at least two independent experiments are plotted; *p<0.05, ***p<0.0001 determined by two-tailed Student's t-test indicate significant difference from control group.

Given the fact that adenosine was able to completely abolish CGS effects on parasite differentiation and also that this drug significantly inhibits adenosine transport in *L. amazonensis* promastigotes, we decided to investigate the role of adenosine in the differentiation of metacyclic promastigotes in further detail. It has been extensively characterized that appearance of metacyclic promastigotes in *Leishmania* cultures occur after the culture reaches the stationary phase of growth, indicating that lack of nutrients would trigger metacyclogenesis. In order to show that lack of purines in the culture medium would be the cause of growth arrest and induction of metacyclogenesis, we harvested metacyclic promastigotes from CGS-treated cultures by density gradient centrifugation and incubated these cells in “spent” culture medium (medium obtained from late stationary phase of promastigotes cultures). As shown in Figure 4A metacyclic promastigotes were not able to proliferate under these conditions. However, addition of adenosine to the spent medium restored parasite proliferation in a dose dependent manner, indicating that adenosine was the lacking nutrient that hampered parasite multiplication. To eliminate the possibility that contaminating non-metacyclic promastigotes were proliferating in the previous experiment, we decided to further purify the metacyclic promastigotes by submitting them to complement mediated lysis after the density gradient centrifugation. In addition, to exclude the possible role of unknown components present in fetal bovine serum, we incubated the parasites in Grace's insect medium (which is devoid of purines) without the addition of any supplement. As shown in Figure 4B, parasite proliferation was observed only in the presence of adenosine. Importantly, proliferation induced by adenosine addition was associated, in the experiments with “spent” medium (data not shown) as well as in experiments without added fetal bovine serum (Figure 4C), with loss of the metacyclic morphology, indicating a reversal of the differentiation process. We did not test other purine nucleosides or nucleobases in these experiments. However, given the ability of adenine and inosine to reverse CGS effects it is possible that these substances would also be able to induce the reversal of metacyclogenesis and induce proliferation.

Finally, in order to validate our *in vitro* results, we decided to evaluate the role of adenosine in *in vivo* metacyclogenesis of *Leishmania infantum chagasi* in *Lutzomyia longipalpis* sand flies. Our results (Figures 4D–F) show that sand flies that were fed a mixture of sucrose and adenosine harbored similar number of promastigotes but significantly less metacyclic forms after a blood meal on a *L. chagasi* naturally infected dog than those fed control sugar solution. This result strongly supports the role of adenosine (and possibly other purine nucleosides) on the control of parasite differentiation in *Leishmania* promastigotes and extends the *in vitro* findings to other parasite species (also see Figure S3).

Our results indicate that absence of purines may have an important role in triggering metacyclogenesis in *Leishmania*. We also show that metacyclogenesis and parasite proliferation are not necessarily associated, although in the invertebrate host, the conditions for metacyclogenesis may coincide with those that will eventually lead to proliferation arrest. *Leishmania* promastigotes proliferate intensely, as procyclic forms, in the insect midgut inside the peritrophic membrane during the digestion of an infected blood meal. After passage of the blood meal, several morphological stages such as nectomonads, leptomonads and metacyclic forms are present [2], [18]. At this point, the sand fly feeds mainly from plant sap which is constituted basically from sucrose, mineral salts, amino acids and proteins [39], [40]. We postulate that the absence of purines in the insect midgut after passage of the blood meal is responsible for triggering metacyclogenesis.

The molecular nature of this process is currently unknown, however, it has been suggested that a “purine sensor” may exist in *Leishmania* [41], [42]. We propose that in conditions of purine deficiency this putative “purine sensor” would trigger metacyclogenesis. In our study, CGS could bind to this “sensor” and inhibit purine detection thus inducing differentiation into metacyclic promastigotes.

As mentioned before, other studies have identified other conditions in which metacyclogenesis is increased [2], [4], [20], [43]. Our study describes a new level of the parasite-vector interaction mediated by a purine sensing mechanism that regulates parasite differentiation and prepares the parasite for the infection of the mammalian host. The existence of a purine sensor that controls parasite differentiation deserves further investigation since it may play an important role in other stages of parasite life cycle, thus revealing new targets for the design of drugs for disease control.

### Accession numbers

Acession number of META-1 protein is available from GenBank (http://www.ncbi.nlm.nih.gov/genbank) as AAC04758.1.

## Supporting Information

Figure S1 **Persistent presence of CGS 15943 in** ***Leishmania amazonensis*** **culture is needed for interruption of parasite growth independently of the time of addition to culture.** Evaluation of *L. amazonensis* (IFLA/BR/67/PH8) growth in Grace's insect medium plus 10% FBS, 2 mM glutamine, 100 IU/ml penicillin, pH 6.5 containing CGS 15943 (50 µM) as determined by hemocytometer counting. Parasites were left for 24 hr in contact of CGS (“for 24 hr”), washed and transferred to fresh medium without CGS. In the “after 24 hr” group, CGS was added to culture after 24 hr of normal growth. Arrow on graph indicates the moment when CGS was added or removed from culture. Means and standard deviations from three independent experiments are plotted; *p<0.05 determined by student's t-test indicate significant difference from control group.
(TIF)

Figure S2 **Presence of purines blocks metacyclogenesis in normal cultures.** Parasites were grown in normal culture medium in the presence of added adenosine (Ado), inosine (Ino), adenine (Ade) or hypoxanthine (Hyp). Purines were added to culture after 48 hr of growth and metacyclic promastigotes quantified after 24 hr by Ficoll density gradient. Means and standard deviations from three independent experiments are plotted; *p<0.05 determined by student's t-test indicate significant difference from control group.
(TIF)

Figure S3 **Presence of CGS 15943 in** ***Leishmania major*** **culture halts growth and enhance metacyclogenesis.** (A) Growth evaluation determined by hemocytometer counting during 72 hr of *L. major* (MHOM/IL/80/Friedlin) in medium (see Figure S1) containing CGS (50 µM). Metacyclogenesis in *L. major* cultures was evaluated by Ficoll density gradient (B). In experiments for metacyclogenesis evaluation, CGS was added to culture after 48 hr of growth and metacyclic promastigotes quantified 24 hr afterwards, as described in Figure 1. Means and standard deviations from three independent experiments are plotted; *p<0.05, determined by student's t-test indicate significant difference from control group.
(TIF)

Video S1 **General aspect of** ***Leishmania amazonensis*** **promastigotes cultivated in Grace's insect medium.** Representative 10 seconds video, made at endpoint of parasite culture (72 hr). This movie shows the general aspect of *L. amazonensis* promastigotes incubated for 72 hr (initiated at 2×10^6^ cells/ml) in Grace's insect medium supplemented with 10% FBS, 2 mM glutamine and 100 U/ml penicillin, pH 6,5.
(MP4)

Video S2 **General aspect of** ***Leishmania amazonensis*** **promastigotes cultivated in Grace's insect medium plus Dimethil Sulfoxide (1%).** Representative 10 seconds video, made at endpoint parasite culture (72 hr). This movie shows the general aspect of *L. amazonensis* promastigotes incubated for 72 hr (initiated at 2×10^6^ cells/ml) in Grace's insect medium supplemented with 10% FBS, 2 mM glutamine and 100 U/ml penicillin, pH 6.5. DMSO was added to cultures after 48 hr of growth and the video was captured 24 hr afterwards. Is important to note that the addition of DMSO does not alter the general appearance (morphology, motility and quantity) of the cells.
(MP4)

Video S3 **General aspect of** ***Leishmania amazonensis*** **promastigotes cultivated in Grace's insect medium plus CGS.** Representative 10 seconds video, made at endpoint parasite culture (72 hr). This movie shows the general aspect of *L. amazonensis* promastigotes incubated for 72 hr (initiated at 2×10^6^ cells/ml) in Grace's insect medium supplemented with 10% FBS, 2 mM glutamine and 100 U/ml penicillin, pH 6.5. CGS (50 µM) was added to cultures after 48 hr of growth and the video was captured 24 hr afterwards. After 24 hr incubated with CGS, culture of *L. amazonensis* showed differences in promastigotes characteristics, especially with regard to cell size and motility. Note the fast moving small parasites.
(MP4)
