# Supplementary figures and images for: Leishmania Metacyclogenesis Is Promoted in the Absence of Purines
Source: PLoS Negl Trop Dis. 2012 Sep 20;6(9):e1833. doi: 10.1371/journal.pntd.0001833 (PMC3458635; doi:10.1371/journal.pntd.0001833)

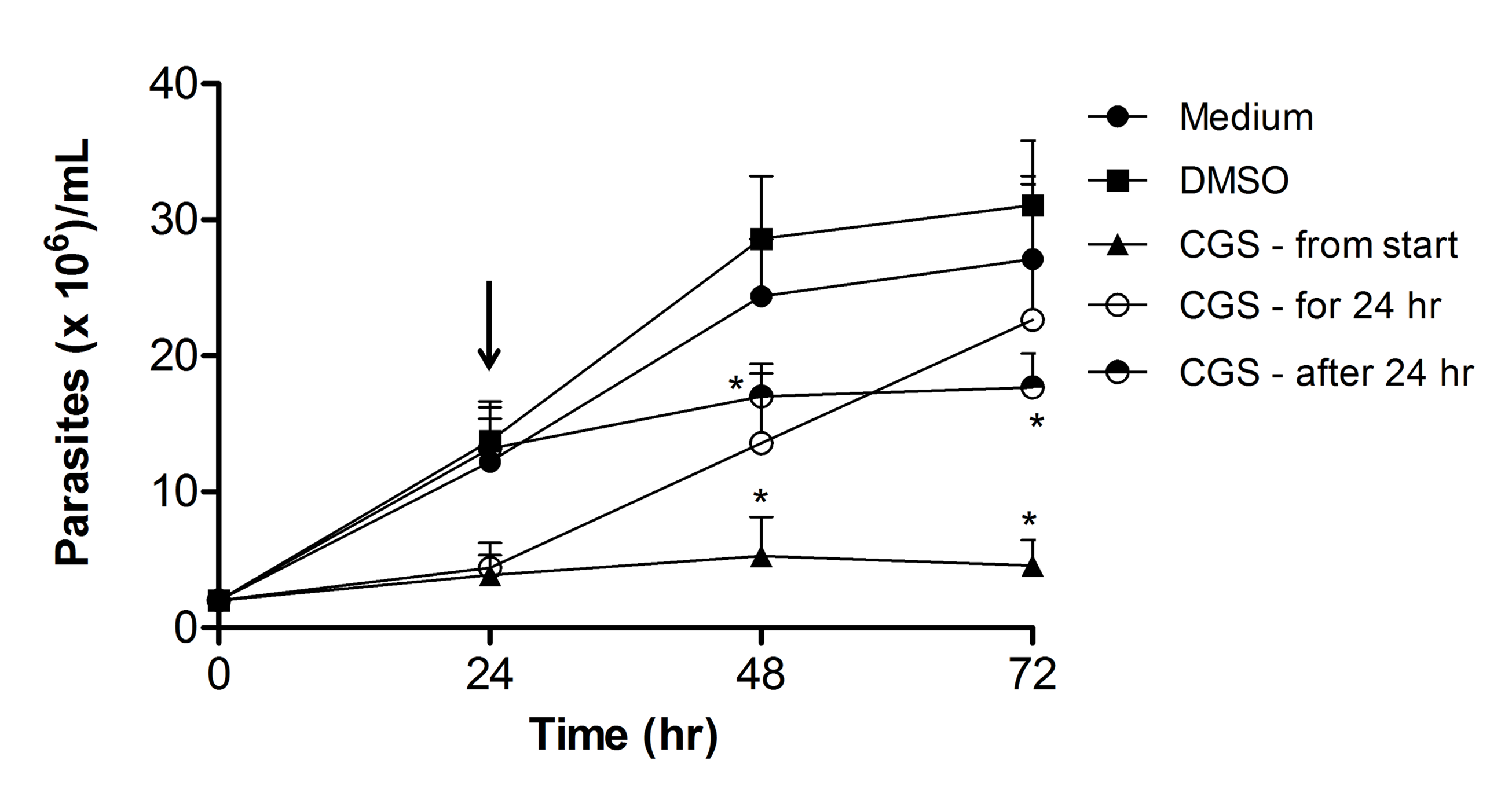

Supplement: Figure S1 — Persistent presence of CGS 15943 in Leishmania amazonensis culture is needed for interruption of parasite growth independently of the time of addition to culture. Evaluation of L. amazonensis (IFLA/BR/67/PH8) growth in Grace's insect medium plus 10% FBS, 2 mM glutamine, 100 IU/ml penicillin, pH 6.5 containing CGS 15943 (50 µM) as determined by hemocytometer counting. Parasites were left for 24 hr in contact of CGS (“for 24 hr”), washed and transferred to fresh medium without CGS. In the “after 24 hr” group, CGS was added to culture after 24 hr of normal growth. Arrow on graph indicates the moment when CGS was added or removed from culture. Means and standard deviations from three independent experiments are plotted; *p<0.05 determined by student's t-test indicate significant difference from control group. (TIF) [file pntd.0001833.s001.tif]

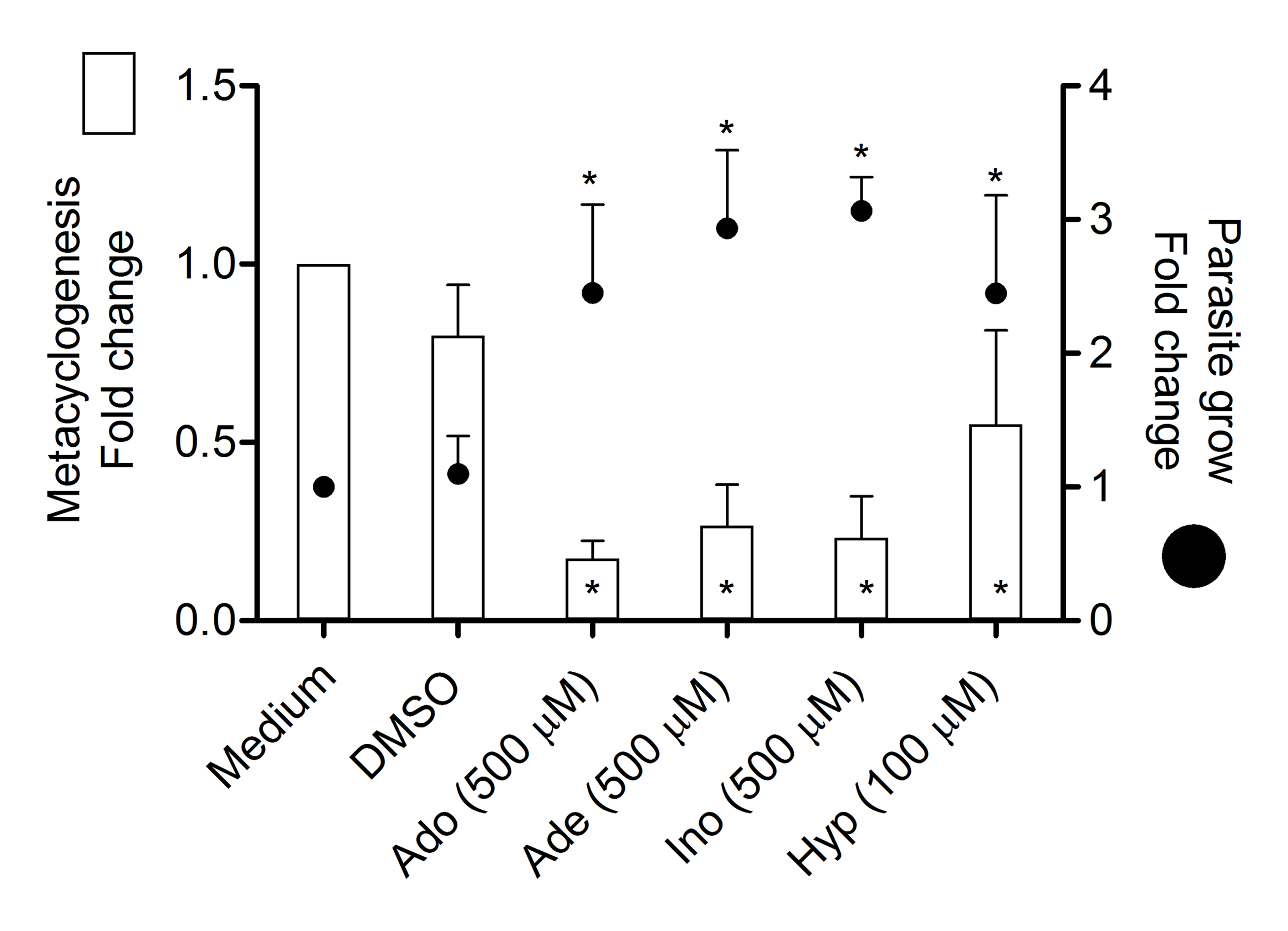

Supplement: Figure S2 — Presence of purines blocks metacyclogenesis in normal cultures. Parasites were grown in normal culture medium in the presence of added adenosine (Ado), inosine (Ino), adenine (Ade) or hypoxanthine (Hyp). Purines were added to culture after 48 hr of growth and metacyclic promastigotes quantified after 24 hr by Ficoll density gradient. Means and standard deviations from three independent experiments are plotted; *p<0.05 determined by student's t-test indicate significant difference from control group. (TIF) [file pntd.0001833.s002.tif]

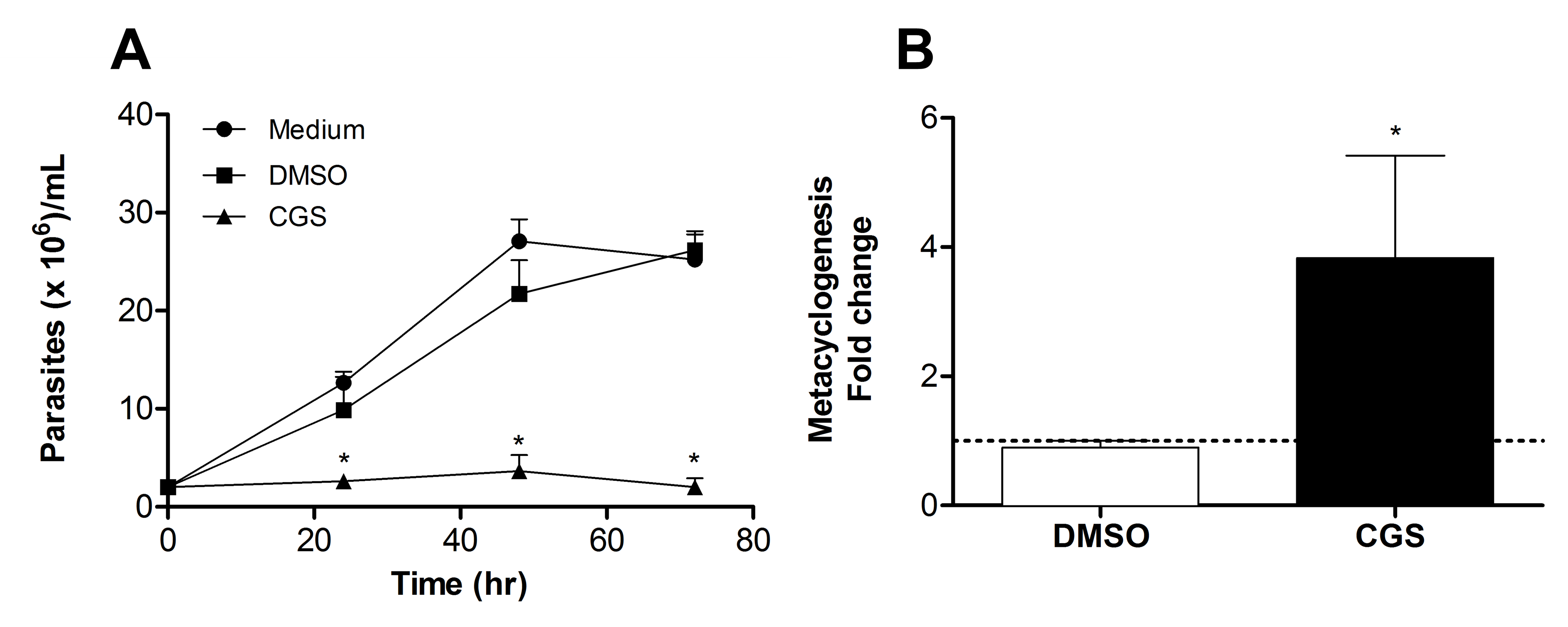

Supplement: Figure S3 — Presence of CGS 15943 in Leishmania major culture halts growth and enhance metacyclogenesis. (A) Growth evaluation determined by hemocytometer counting during 72 hr of L. major (MHOM/IL/80/Friedlin) in medium (see Figure S1) containing CGS (50 µM). Metacyclogenesis in L. major cultures was evaluated by Ficoll density gradient (B). In experiments for metacyclogenesis evaluation, CGS was added to culture after 48 hr of growth and metacyclic promastigotes quantified 24 hr afterwards, as described in Figure 1. Means and standard deviations from three independent experiments are plotted; *p<0.05, determined by student's t-test indicate significant difference from control group. (TIF) [file pntd.0001833.s003.tif]
